# Genome-wide variation in recombination rate in *Eucalyptus*

**DOI:** 10.1186/s12864-016-2884-y

**Published:** 2016-08-09

**Authors:** Jean-Marc Gion, Corey J. Hudson, Isabelle Lesur, René E. Vaillancourt, Brad M. Potts, Jules S. Freeman

**Affiliations:** 1 CIRAD, UMR AGAP, 69 route d’Arcachon, Cestas, France; 2 School of Biological Sciences, University of Tasmania, Private Bag 55, Hobart, TAS 7001 Australia; 3 Present address: Tasmanian Alkaloids, P.O. Box 130, Westbury, TAS 7303 Australia; 4 HelixVenture, Merignac, F33700 France

**Keywords:** *Eucalyptus globulus*, Meiotic recombination, Crossover, Genomic features, Genetic diversity, Gene density

## Abstract

**Background:**

Meiotic recombination is a fundamental evolutionary process. It not only generates diversity, but influences the efficacy of natural selection and genome evolution. There can be significant heterogeneity in recombination rates within and between species, however this variation is not well understood outside of a few model taxa, particularly in forest trees. Eucalypts are forest trees of global economic importance, and dominate many Australian ecosystems. We studied recombination rate in *Eucalyptus globulus* using genetic linkage maps constructed in 10 unrelated individuals, and markers anchored to the *Eucalyptus* reference genome. This experimental design provided the replication to study whether recombination rate varied between individuals and chromosomes, and allowed us to study the genomic attributes and population genetic parameters correlated with this variation.

**Results:**

Recombination rate varied significantly between individuals (range = 2.71 to 3.51 centimorgans/megabase [cM/Mb]), but was not significantly influenced by sex or cross type (F_1_ vs. F_2_). Significant differences in recombination rate between chromosomes were also evident (range = 1.98 to 3.81 cM/Mb), beyond those which were due to variation in chromosome size. Variation in chromosomal recombination rate was significantly correlated with gene density (*r* = 0.94), GC content (*r* = 0.90), and the number of tandem duplicated genes (*r* = −0.72) per chromosome. Notably, chromosome level recombination rate was also negatively correlated with the average genetic diversity across six species from an independent set of samples (*r* = −0.75).

**Conclusions:**

The correlations with genomic attributes are consistent with findings in other taxa, however, the direction of the correlation between diversity and recombination rate is opposite to that commonly observed. We argue this is likely to reflect the interaction of selection and specific genome architecture of *Eucalyptus*. Interestingly, the differences amongst chromosomes in recombination rates appear stable across *Eucalyptus* species. Together with the strong correlations between recombination rate and features of the *Eucalyptus* reference genome, we maintain these findings provide further evidence for a broad conservation of genome architecture across the globally significant lineages of *Eucalyptus*.

## Background

Meiotic recombination is a fundamental evolutionary process, which is believed to have been central to the evolutionary success of eukaryotes. When crossover recombination occurs, DNA strands are exchanged between homologous chromosomes, resulting in allele shuffling. This generates novel genetic variation [1], by breaking the associations that occur both within and between linked genes [2]. As this genetic variation is sieved by natural selection, it shapes the adaptive evolution of living organisms and thus, indirectly, genome evolution.

Aside from allele shuffling, recent studies have identified four other processes by which recombination directly influences genome evolution [1, 3]. These are: GC-biased gene conversion, whereby recombination promotes GC enrichment at repair sites; Hotspot drive, through which a higher transmission of non-recombinant alleles likely contributes to rapid shifts in the location of recombination hotspots [4, 5]; through the potential Mutagenic effect of recombination itself, which causes structural mutations; and Indel drive, a neutral mechanism that may drive genome contraction by a higher transmission of chromosomal deletions compared to insertions [6, 7]. In combination, these processes enable recombination to substantially influence genome evolution at a variety of genomic scales.

Historically, recombination was studied through observation of chiasmata using microscopy [8], or by genetic linkage mapping using phenotypic markers. More recently, recombination has been studied through variation in recombination rate (RR); estimated from genetic distance (i.e., number of recombination events) divided by physical distance (the size in base pairs of DNA over which the genetic distance is measured) using three main approaches, linkage disequilibrium mapping, sperm typing and linkage mapping using molecular markers. The major differences between these approaches are whether they measure historical or current recombination and their applicability to populations, a single, or few individuals [9]. Linkage disequilibrium mapping estimates historical recombination at the population level, and is thus useful for understanding population evolution, but is affected by factors such as genetic drift, demography, mutation rate and natural selection. In contrast, both sperm typing and linkage mapping are little affected by these factors and provide direct estimates of current recombination across a single generation [9]. Linkage mapping is a classic technique widely used to study recombination in selfed or bi-parental families. Direct estimates of RR can be obtained from the genetic distance between pairs of linked markers compared to their physical distance. The resolution of linkage mapping studies is limited by sample size and the density of molecular markers. However, the use of high-throughput marker systems and whole genome sequences potentially allows fine scale study of RR, as well as comparison between sexes, populations and individuals [10].

Understanding variation in RR will contribute to a better understanding of genome evolution and is fundamental to many aspects of genetics. For example, knowing the genomic distribution of RR is important for predicting the potential of populations to respond to environmental change, as well as for breeding using both traditional and genomic approaches. Specifically, characterising RR will help predict the degree of linkage disequilibrium to guide marker densities needed for genomic selection, a technique which is showing promise for both animal and plant breeding [11]. It also has practical applications when trying to isolate the specific genes underlying quantitative trait loci (QTL) that have been detected in linkage mapping or association genetic studies, and for attempts to introduce favourable alleles into breeding lines [12, 13].

Most detailed studies of recombination and linkage disequilibrium have been accomplished in human, yeast, *Drosophila* or *Arabidopsis*, which have the required genomic resources (such as good reference genomes and high throughput marker systems). Although many of the factors which influence RR are conserved across eukaryotic organisms, empirical studies consistently report variation in RR between taxa, individuals and across genomes [9, 10, 14]. In view of this variation, it is important to obtain a broad understanding of RR across diverse taxa. This is particularly true for long-lived organisms such as forest trees which often have many genetic features, including low rates of selfing, high heterozygosity and high population genetic diversity [15, 16] which distinguish them from most model species. Trees often define and structure forest ecosystems; play vital ecosystem services, including climate-change mitigation; provide invaluable products; and are widely used for human recreation. Given their environmental and economic significance, understanding the genetic mechanisms which shape the evolution and diversity of trees is of major importance.

The genomic resources required to study RR have only recently become available in forest trees [17–19] thus there is little knowledge of the genomic distribution of RR in these organisms. Nonetheless, insights into the patterns of recombination in tree species have been gained from studies of linkage disequilibrium (e.g., Slavov et al. [20], reviewed by Thavamanikumar et al. [21]) and comparative mapping [22]. Assembled reference genomes are available for hardwood forest trees, such as *Populus* [17], *Eucalyptus* [19], *Quercus* [23] and *Castanea* [24] providing the opportunity to directly estimate genome-wide RR using linkage maps. Recent studies in *Eucalyptus* have compared genetic to physical map distance and reported RR averaged over the whole genome [25] or for each chromosome [26, 27]. Silva-Junior and Grattapaglia [27] also calculated high resolution estimates of linkage disequilibrium and ‘population scaled recombination’ rate (an estimate of the history of recombination in different genomic segments), and its genomic correlates. However, for direct estimates of RR there is little information regarding variation between individuals, factors influencing this variation, nor its correlation with genomic features in forest trees.

*Eucalyptus* is a diverse genus containing over 700 species across thirteen subgenera [28]. Most species belong to subgenus *Symphyomyrtus* including the majority of economically important plantation species [29], and the two species used in this study; *E. grandis* (section *Latoangulatae*) and *E. globulus* (section *Maidenaria*) [28]. Eucalypts generally have a mixed-mating system (i.e., they can reproduce both by selfing and outcrossing), and are mostly animal pollinated. Their outcrossing rate is variable, but generally high and most species are highly polymorphic [29, 30]. For example, recent findings indicate genome-wide diversity in *E. grandis* [27] is at the high end for plants, in line with other angiosperm trees such as *Populus trichocarpa* and an order of magnitude higher than most conifers, corroborating previous findings in eucalypt [19, 29]. All eucalypt species studied to date possess the same chromosome number (n = 11), although their genomes vary in size from 360 Mb (*Corymbia variegata*) to 640 Mb (*Eucalyptus grandis*) [29]. Despite this variation in genome size, there is a high degree of karyotype stability between species [29], which includes broad conservation of chromosome structure such as the location of centromeres and heterochromatin content within the subgenus *Symphyomyrtus* [31], and this is supported by comparative mapping studies [32, 33]. Specifically, there was no evidence for gross chromosomal rearrangements between (the 530 Mb genome of) *E. globulus* and an *E. grandis/urophylla* consensus map based on nearly 400 shared markers [33]. Re-sequencing of the *E. globulus* and *E. grandis* genomes suggested that the differences in genome size between these species are largely attributable to many small changes distributed throughout their genomes [19]. Further, the genomic distribution of diversity within and divergence between eucalypt species in subgenus *Symphyomyrtus,* appears stable and correlated with genomic architecture [34], providing further evidence for genome stability. In view of this stability, the numbering of chromosomes in most recent molecular studies in eucalypt has followed the consensus linkage map of Brondani et al. [35], and this order was used for the 11 major scaffolds in the *E. grandis* genome [19].

We here focus on *Eucalyptus globulus*, and exploit the recently published *Eucalyptus grandis* reference genome in conjunction with 10 linkage maps constructed with sequenced anchored markers. This experimental design allowed testing of the effects of various factors reported in other species to be associated with chromosomal variation in RR, including chromosome, sex, and cross-type; genomic features such as GC content, density of genes and transposable elements; and the population genetic parameters, genetic diversity and divergence (calculated from six species from subgenus *Symphyomyrtus*, [34]; see methods for detail).

## Results

### Variation between individuals

**Fig. 1:**
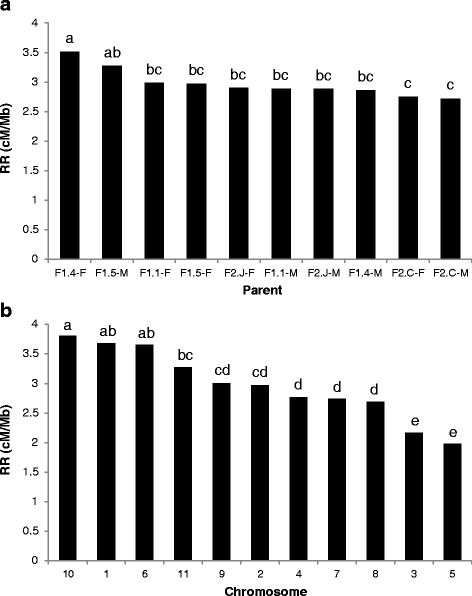
Variation in recombination rate between individuals and chromosomes in *Eucalyptus globulus.* **a** Mean genome-wide RR for each of the 10 *Eucalyptus globulus* individuals. **b** Mean RR in each chromosome across the 10 individuals. Means with different letters were significantly different in pairwise comparisons following the Tukey-Kramer (honestly significant difference, HSD) adjustment

The difference in RR amongst parents (Fig. 1a) was highly significant (F_9,90_ = 6.1, *P* < 0.001) and due mainly to the high RR of two F_1_ parents (F1.4-F and F1.5-M) and low RR in the male and female parents of one of the F_2_ families (F2.C). The RR of the F_1_ families (3.1 ± 0.09 cM/Mb) was greater than that of the F_2_ families (2.8 ± 0.11 cM/Mb), but not significantly so (F_1,8_ = 3.9, *P* = 0.085). The F_2_ families had higher levels of heterozygosity than the F_1_ (2-tailed *t*_8_ = 7.04, *P* < 0.001). However while negative, the correlation between parental heterozygosity and RR was not significant. There was no significant differences in RR between sexes (F_1,8_ = 0.39, *P* = 0.549). The genome-wide RR ranged between 2.71 and 3.51 cM/Mb between the 10 *E. globulus* parents, and averaged 2.98 cM/Mb (Table 1). These values were calculated across linkage maps ranging in size (i.e., genetic distance, estimated by Haldane’s mapping function) from 1442 to 1787 cM (Table 2), averaging 1545 cM.

**Table 1:** Chromosome and genome scale descriptive statistics^a^ of the data used to study recombination in *Eucalyptus*

Level and variable	Min	Max	Mean	CV^e^
*Chromosome averages (n = 11)*				
Physical size (Mb)	39.02	80.10	55.10	0.29
Diversity (global H_Hw_)^b^	0.196	0.229	0.196	0.04
Divergence (global F_*st*_)^c^	0.385	0.405	0.354	0.03
GC content (%)	35.60	38.29	37.23	0.02
RR (cM/Mb)	1.98	3.81	2.98	0.20
*Individual genome-wide averages (n = 10)*				
Genetic distance (cM)	1442	1787	1545	0.08
Physical size (Mb)	508.3	569.5	543.1	0.03
Genome coverage (%)	84.13	93.55	89.37	0.03
Heterozygosity^d^	630	815	708	0.10
RR (cM/Mb)	2.71	3.51	2.98	0.08

^a^Diversity and divergence were estimated by Hudson et al. [34]. The remaining variables were estimated from our *E. globulus* data and the *E. grandis* genome [19]

^b^The diversity within 6 *Eucalyptus* species, including *E. globulus*

^c^Divergence between the 6 *Eucalyptus* species

^d^Estimated from the total number of polymorphic markers derived from the DArT assay (see Methods)

^e^Coefficient of variation

**Table 2:** Detailed summary of the linkage maps used in this study

		Scaffold ID	1	2	3	4	5	6	7	8	9	10	11
Cross	P^a^	Scaffold Size (Mb)^b^	40.3	64.2	80.1	42.0	74.7	53.9	52.4	74.3	39.0	39.3	45.5
F1.1	F	Interval Number	8	8	15	6	17	10	12	11	10	5	8
		Total length (cM)	122	170	174	99	125	199	147	206	132	91	122
		Phys. Size (Mb)	36.1	58.0	67.6	40.6	71.0	52.6	51.7	64.7	37.2	26.5	41.5
		Mean RR (cM/Mb)	3.38	2.93	2.57	2.44	1.76	3.78	2.84	3.19	3.55	3.44	2.94
	M	Interval Number	7	10	16	8	11	10	9	14	8	4	9
		Total length (cM)	150	176	159	82	129	173	105	128	99	113	139
		Phys. Size (Mb)	38.1	54.8	73.0	34.1	72.3	49.2	41.8	56.4	35.1	29.1	42.3
		Mean RR (cM/Mb)	3.93	3.21	2.18	2.40	1.78	3.52	2.51	2.27	2.82	3.88	3.29
F1.4	F	Interval Number	8	9	19	6	16	9	13	13	8	3	12
		Total length (cM)	176	199	184	98	145	183	180	224	118	117	163
		Phys. Size (Mb)	37.4	55.3	75.8	36.0	72.3	45.3	49.9	59.3	34.3	25.4	43.9
		Mean RR	4.71	3.60	2.43	2.72	2.01	4.04	3.61	3.77	3.44	4.61	3.72
	M	Interval Number	9	10	17	4	12	8	12	16	6	7	16
		Total length (cM)	153	144	142	110	143	154	115	177	82	99	137
		Phys. Size (Mb)	39.3	51.8	74.5	34.9	66.3	38.9	51.2	71.9	33.5	31.0	41.5
		Mean RR (cM/Mb)	3.90	2.78	1.91	3.15	2.16	3.96	2.25	2.46	2.45	3.19	3.30
F1.5	F	Interval Number	7	7	17	7	10	9	11	14	9	9	11
		Total length (cM)	115	142	136	101	109	165	152	170	104	104	144
		Phys. Size (Mb)	34.4	47.9	67.9	35.6	59.4	40.2	51.9	65.3	36.1	27.6	42.1
		Mean RR (cM/Mb)	3.35	2.96	2.00	2.84	1.84	4.11	2.93	2.60	2.88	3.77	3.42
	M	Interval Number	6	14	12	7	12	11	8	10	7	11	11
		Total length (cM)	139	157	184	116	166	181	173	206	109	136	151
		Phys. Size (Mb)	31.6	56.8	71.6	35.7	71.0	52.6	51.1	66.4	36.7	32.1	42.3
		Mean RR (cM/Mb)	4.40	2.76	2.57	3.25	2.34	3.44	3.38	3.10	2.97	4.24	3.57
F2.J	F	Interval Number	6	13	17	8	14	11	10	15	9	10	10
		Total length (cM)	107	167	139	101	129	163	101	158	105	162	168
		Phys. Size (Mb)	36.1	56.0	76.1	36.7	69.7	43.3	47.2	66.4	34.8	36.7	43.4
		Mean RR (cM/Mb)	2.96	2.98	1.83	2.75	1.85	3.76	2.14	2.38	3.02	4.42	3.87
	M	Interval Number	10	9	17	12	13	14	14	15	8	14	16
		Total length (cM)	129	165	153	92	141	164	142	181	121	130	124
		Phys. Size (Mb)	36.1	55.0	75.4	32.3	72.9	53.1	52.3	71.7	37.5	33.2	42.7
		Mean RR (cM/Mb)	3.57	3.00	2.03	2.85	1.93	3.09	2.71	2.52	3.23	3.92	2.90
F2.C	F	Interval Number	5	13	16	9	14	15	10	14	11	9	8
		Total length (cM)	105	164	160	110	144	168	133	137	117	120	118
		Phys. Size (Mb)	35.0	61.1	69.7	38.0	67.0	52.2	51.1	64.7	38.0	35.0	42.3
		Mean RR (cM/Mb)	3.00	2.68	2.30	2.90	2.15	3.22	2.60	2.12	3.08	3.43	2.79
	M	Interval Number	6	11	18	11	12	12	14	17	8	8	12
		Total length (cM)	119	172	139	92	136	189	121	178	102	113	124
		Phys. Size (Mb)	32.6	61.5	76.2	39.1	69.4	52.0	50.3	72.3	38.3	35.6	42.3
		Mean RR (cM/Mb)	3.65	2.80	1.82	2.35	1.96	3.64	2.41	2.46	2.66	3.18	2.93

^a^P = Parent, F = Female, M = Male ^b^Phys. Size = Physical Size

### Variation between chromosomes

The 11 chromosomes of *E. globulus* differed markedly in RR. Averaged across parents, chromosome-wide RR ranged from 1.98 to 3.81 cM/Mb (Table 1). The difference between chromosome averages was highly significant when tested using parents as replicates (F_10,90_ = 34.0, *P* < 0.001), and was also significant (F_10,89_ = 92.7, *P* < 0.001) after removing the correlated effect of chromosome size (see discussion below). The variation between chromosomes was mainly due to the high RR in chromosomes 10, 1 and 6 and low RR in chromosomes 3 and 5 (Fig. 1b). The difference between chromosomes was moderately to highly positively correlated with previously published estimates for *E. grandis x urophylla* consensus maps ([26] *n* = 11, Pearson *r* = 0.90, *P* < 0.001; [25] *n* = 11, Pearson *r* = 0.92, *P* < 0.001) as well as parental maps [27] of *E. grandis* (*n* = 11, Pearson *r* = 0.72, *P* = 0.012) and *E. urophylla* (*n* = 11, Pearson *r* = 0.55) although the latter was not significant (*P* = 0.081). In no case was the interaction of chromosome with either sex (F_10,80_ = 1.17, *P* = 0.327) or cross type (F_10,80_ = 1.03, *P* = 0.426) significant, indicating that the chromosomal differences in RR were stable across sex and cross-type in this study.

Several genomic features of the *E. grandis* reference genome were correlated with the chromosomal variation in RR (Table 3). Chromosomes with high RR were smaller, had higher GC content, higher gene density, lower frequencies of several categories of transposable elements and fewer tandemly duplicated genes (Table 4). While the association with transposable elements as a whole was not significant (*r* = −0.59, *P* = 0.056), significant negative correlations were observed for specific classes of transposable elements (DNA transposable elements *r* = −0.76, *P* = 0.007 and uncategorised elements, i.e., those that could not be assigned to any specific transposable element class, *r* = −0.97, *P* < 0.001), and superfamilies within both the retrotransposons (long interspersed nuclear elements/short interspersed nuclear elements [LINE/SINE] *r* = −0.78, *P* = 0.005) and DNA transposable elements (helitrons *r* = −0.69, *P* = 0.019). Chromosomes with high RR also tended to have a lower density of tandem gene duplications (*r* = −0.72, *P* = 0.012). However, most of these genomic features were inter-correlated (Table 3) and when the effect of gene density (the most highly correlated feature in Table 3) was removed by calculation of partial correlation coefficients, correlations between RR and all other factors listed in Table 3 became insignificant. Partialling out the effect of chromosome size had little effect on the correlation between RR and other genome features, and the correlations involving gene density (partial *r* = 0.87, *P* < 0.001) and %GC (partial *r* = 0.76, *P* = 0.010) remained significant.

**Table 3:** Pearson correlations among chromosome attributes (n = 11)

	RR		Chromosome length		GC content		Gene density		Transposable element density		Number of tandem duplicated genes		Diversity	
Chromosome length (bp)	−0.74	**												
GC content (%)	0.90	***	−0.76	**										
Gene density	0.94	***	−0.70	*	0.91	***								
Transposable element density	−0.59	ns	0.32	ns	−0.29	ns	−0.62	*						
Number of tandem duplicated genes	−0.72	*	0.53	ns	−0.70	*	−0.75	**	0.46	ns				
Diversity (Global H_HW_)^a^	−0.75	**	0.61	*	−0.82	**	−0.85	***	0.41	ns	0.78	**		
Divergence (Global F_*st*_)^b^	0.90	***	−0.84	**	0.95	***	0.92	***	−0.40	ns	−0.79	**	−0.91	***

^a^The diversity within 6 *Eucalyptus* species, including *E. globulus*

^b^Divergence between the 6 *Eucalyptus* species

Significance levels are: *** *P* < 0.001, ** *P* < 0.01, * *P* < 0.05, ns *P* ≥ 0.05

**Table 4:** Chromosome mean values for the main attributes used in correlation analyses^a^

Chromosome	Recombination rate (cM/Mb)	Chromosome length (Mbp)^b^	GC content (%)	Gene density^c^	Transposable element density^d^	Number of tandem duplicated genes^e^	Diversity (Global H_HW_)	Divergence (Global Fst)
1	3.68	40.30	0.380	63.55	401.86	0.088	0.181	0.400
2	2.97	64.24	0.364	53.05	392.64	0.092	0.206	0.376
3	2.16	80.09	0.357	42.84	416.24	0.103	0.229	0.354
4	2.77	41.98	0.372	53.03	430.05	0.085	0.188	0.395
5	1.98	74.73	0.353	44.35	418.50	0.100	0.215	0.359
6	3.66	53.89	0.377	71.49	361.55	0.077	0.175	0.400
7	2.74	52.45	0.368	52.70	412.18	0.092	0.219	0.373
8	2.69	74.33	0.375	56.09	439.17	0.090	0.189	0.383
9	3.01	39.02	0.375	61.00	398.21	0.096	0.194	0.391
10	3.81	39.36	0.383	69.77	406.39	0.090	0.188	0.405
11	3.27	45.51	0.377	67.35	389.91	0.088	0.165	0.403

^a^All chromosome attributes are from the *Eucalyptus grandis* reference genome [19], except diversity and divergence which were estimated by Hudson et al. [34]

^b^Equivalent to 'Scaffold Size' in Table 2

^c^ Number of genes per Mbp

^d^ Number of transposable elements per Mbp

^e^ The proportion of annotated genes which are tandem duplicates

Chromosomal variation in RR was also associated with differences in marker diversity (assuming Hard-Weinberg equilibrium [*H*_*Hw*_]) and species divergence (measured as the proportion of the genetic variance in a sub population relative to the total genetic variance [*F*_*st*_]). It was highly negatively correlated with the pooled genetic diversity (global *H*_*Hw*_) within the six *Eucalyptus* species studied by Hudson et al*.* [34] (*n* = 11, *r* = −0.75, *P* = 0.008). The correlation between chromosomal RR and *H*_*Hw*_ of each individual species, including *E. globulus*, were similar in size and all also negative (*r* = −0.60 to −0.74)*.* The trends in global diversity were also evident regardless of whether markers were intra- or intergenic. However, for global *H*_*Hw*_ the correlation was only significant for intragenic markers (*r* = −0.65, *P* = 0.032). The global *F*_*st*_ amongst the six species studied by Hudson et al*.* [34] was positively correlated with RR at the chromosome level (*r* = 0.90, *P* < 0.001). This relationship with *F*_*st*_ was also found for both intragenic (*r* = 0.93, *P* < 0.001) and intergenic (*r* = 0.75 *P* = 0.008) markers.

## Discussion

This is the first study in forest trees to present direct, genome-wide, estimates of RR from multiple mapping pedigrees. We show RR varies between individuals as well as between chromosomes. The relative differences amongst chromosomes appear stable across eucalypt species, and are correlated with genomic features commonly related to RR in diverse taxa; including GC content, gene density and the density of specific transposable elements. Significantly, we show that the differences in RR are negatively correlated with species-wide genetic diversity at the chromosome level. The direction of this correlation is the opposite to that commonly observed (see reviews by Smukowski and Noor [9], and Cutter and Payseur [36]) and, we argue, is likely to reflect an interaction of selection and genome architecture.

### Variation between individuals

The variation we found in genome-wide RR between individuals is consistent with reports in other studies [9, 37, 38] and may be due to environmental or genetic factors. RR has been shown to be affected by the environment in both plants and animals [39–41]. However, the role of the environment cannot be clarified in the present study as our parents come from different environments as well as being genetically distinct. Genetic factors which may influence RR, include sex and cross type. Sex specific differences have been reported in some taxa, with elevated RR often occurring in the gender subject to gametic selection [42]. However, no difference in RR between sexes was evident in *E. globulus*. There was a signal in our data, albeit not significant, that cross type could explain some of the variation between individuals, with lower average RR in the F_2_’s compared with the F_1_’s. However further study will be required to validate this. Irrespective of whether the genome-wide variation in RR we observed between individuals is due to genetic or environmental factors, it is clear that representative maps of the RR landscape in *Eucalyptus* species will need to integrate results from multiple crosses and individuals.

Our genome-wide average RR for *E. globulus* (2.98 cM/Mb) was higher than estimates from *E. grandis* (2.17 cM/Mb), *E. urophylla* (2.24 cM/Mb) [27], and *E. grandis*/*urophylla* consensus maps (1.58 cM/Mb and 1.95 cM/Mb; [25] and [26], respectively). However, differences in the scale over which RR was estimated is likely to have influenced the different estimates. Differences in genome coverage also no doubt influenced the estimates of RR between studies, particularly in the case of Kullan et al. [25], which estimated RR based on 152 windows of approximately 1 cM across the genome. Direct comparison of the estimates from different studies is also problematic because different mapping techniques produce different genetic distances and thus different estimates of genetic to physical distance. Our estimates of genetic distance (cM) were derived from Joinmap software, using the maximum likelihood algorithm, which uses Haldane’s mapping function. The estimates of genetic distance used by Petroli et al. [26] were derived from RECORD software [43]. RECORD produces distances very similar to the Joinmap maximum likelihood algorithm, if Haldane’s mapping function is used [44]. However, Silva-junior and Grattapaglia [27] used Kosambi’s mapping function, which produces smaller estimates of genetic distance relative to Haldane’s function. Similarly, both Kullan et al. [25] and Petroli et al. [26] used the regression algorithm and Kosambi’s mapping function in Joinmap, which produce considerably smaller genetic distances than Joinmap’s maximum likelihood algorithm. Therefore, the greater estimate of genome-wide RR in our study may simply reflect greater estimates of genetic distance due to methodological issues. Clearly, further study is required to draw robust conclusions regarding genome-wide differences in RR within and between eucalypt species, ideally using linkage maps constructed from similar techniques in multiple representatives of each species.

### Variation between chromosomes

There was substantial variation in RR between chromosomes in our study, indeed chromosome was the most significant explanatory variable in our analysis of variance. Few studies in plants have examined genome-wide RR using multiple crosses, which are needed to provide the replication to test for significant differences between chromosomes. Nonetheless, chromosomal variation in RR has been demonstrated in a range of taxa [45, 46], including plants such as maize in which intraspecific variation was of a similar magnitude to our findings [14]. Chromosomal variation in RR is likely to at least in part reflect variation in chromosome size, due to the requirement for at least one crossover per chromosome per meiosis [45]. However, in our study the variation in RR between chromosomes remained significant after removing the correlated effect of chromosome size, arguing that other aspects of genomic architecture also contribute to this variation.

Despite the significant differences in genome-wide RR between individuals, three lines of evidence suggest that the relative RR in each chromosome is highly stable; not only within *E. globulus*, but between *Eucalyptus* species, and across evolutionary time. First, the differences between chromosomes in *E. globulus* were evident even when the significant effect of variation amongst the individuals was removed; and there was no significant interaction between chromosome and either sex or cross type within *E. globulus*. Indeed, the difference in RR amongst chromosomes was nearly two fold, whereas that amongst individuals was only 1.3 fold. Second, the high positive correlation between our chromosomal estimates of RR from *E. globulus* (section *Maidenaria*) and those in *E. grandis*/*urophylla* (section *Latoangulatae*) [25, 26] suggests the relative chromosomal differences in RR are stable across these *Eucalyptus* sections, despite an estimated 10 to15 Ma since their divergence [47]. Third, a positive correlation between GC content and RR is expected based on studies in other taxa (see below). The high positive chromosome-level correlation between GC content (from the *E. grandis* genome) and RR (measured from *E. globulus* map distance against the *E. grandis* genome), also points to stability of RR between *E. grandis* and *E. globulus*. Further, because our estimate of RR is from a single generation of recombination, while GC content in *E. grandis* reflects historical RR, the high positive correlation also points to a long term stability of RR at the chromosome level.

### Association with genomic features and population genetic parameters

The chromosome level correlation between GC content and RR we observe is consistent with the theory of ‘GC-biased gene conversion’, whereby DNA repair during recombination promotes enrichment in GC bases, accounting for the positive correlation between RR and GC content seen in most eukaryotes studied to date [9, 48]. The particularly strong positive correlation observed between chromosome level RR and GC in *E. globulus* may reflect the unusually high GC content seen in the family Myrtales, compared with other eudicots [48]. In contrast, *Arabidopsis* is relatively GC poor and GC content and RR are uncorrelated, in line with a general pattern of greater GC enrichment in more derived plant genomes [48].

Our results also suggest a strong positive correlation between chromosomal estimates of RR and gene density. This finding is consistent with the positive correlation between RR and gene density observed at the whole-genome level in a range of taxa [9]. In plants such as maize, rice, wheat, and *A. thaliana* much of this correlation is attributable to the low density of genes in recombination-poor heterochromatin [49]. While in vertebrate genomes, Nam and Ellegren [7] hypothesise neutral processes associated with recombination, including a bias in deletions over insertions, contribute to a more compact genome structure and greater gene density in regions of elevated RR. However, the correlation between gene density and RR varies in strength and direction amongst taxa [9, 36, 50]. The positive correlation we observe at the chromosome level is particularly strong relative to other reports. Consistent with our findings, gene density was positively correlated with historical recombination rate in *E. grandis* (i.e., population scaled recombination rate, measured in 100 kb windows across the genome; [27]), suggesting the relationship between gene density and RR may be widespread in *Eucalyptus*, at least across the subgenus *Symphyomyrtus*.

A negative correlation between RR and some categories of transposable elements was also evident in our study. Transposable elements comprise over 50 % of most eukaryotes genomes and have a significant impact on genome evolution [51, 52]. In particular, it is well accepted that the accumulation of transposable elements is a major factor driving genome expansion [53]. The relative density of transposable elements across the genome reflects a balance between transposable element insertion and deletion. Transposable element deletion is believed to be primarily driven by recombinational processes, consistent with the observed transposable element accumulation in areas of low recombination in many taxa [49, 54] and in line with the correlation we observe. The mechanisms contributing to transposable elements accumulation in regions of low RR are the subject of some debate. Leading hypothesis involve relaxation of various forms of selection in these regions (see Dolgin and Charlesworth [55]); biased insertion of transposable elements in areas of low RR [56]; and a general deletion bias associated with recombination [7]. Regardless of the causative factors, in our study it is possible that in chromosomes with relatively low RR, a greater density of transposable elements has contributed to a reduced gene density, and thus contributed to the positive correlation we observe between RR and gene density at the chromosome level.

A notable finding to emerge from this study is our strong negative correlation between RR at the chromosome-level and species-wide genetic diversity. The common trend in animals is for a positive association between RR and genetic diversity, whereas the association in plants is more variable, but generally zero or positive [36]. The relationship between RR and genetic diversity may be affected by the scale and type of measures of RR (e.g., chromosome averages versus smaller genomic regions) and genetic diversity (i.e., within individuals, populations or species) both of which differ widely between studies [9]. Nevertheless, only in *Oryza* species (wild and domesticated rice) have negative correlations between RR and genetic diversity been previously reported [36]. In *O. sativa*, this association involved 100 kb intervals across three chromosomes (*r* = −0.183 and −0.314; for *O. sativa* ssp. *indica* and ssp. *japonica*, respectively) [57].

The more commonly observed positive correlation between RR and genetic diversity is often attributed to the impact of linked selection [9, 36]. Linked selection predicts a positive relationship between RR and genetic diversity due to the homogenising effects of selection being spread further in regions of low recombination [58]. However, as noted above there is disparity in this relationship between species [36] suggesting other factors also influence how selection impacts the genomic distribution of diversity, such as gene density [59]. Gene dense regions will be on average subject to greater selection, reducing diversity. Therefore, a more general prediction for the effect of selection across genomes is that the distribution of genetic diversity will be related to the density of elements under selection, i.e., gene density (assuming genes are the most likely targets of selection) [59]. This was the case in a recent study of *E. grandis*, where nucleotide diversity (at the population level) was negatively correlated with gene density in 100 kb windows across the *E. grandis* genome; and amongst the genomic features analysed, gene density yielded the highest correlation with nucleotide diversity, far exceeding the strength of the correlation between historical recombination rate and nucleotide diversity [27].

Thus when gene density and RR are strongly positively correlated, as in *E. globulus*, this may counter the influence of RR on linked selection, disrupting the relationship between RR and genetic diversity [36]. Indeed, there is growing evidence that the effects of linked selection are not confined to areas of low recombination but are often pervasive throughout genomes [36, 60], including in the forest tree poplar [61]. In support of this hypothesis, many plants exhibit a positive correlation between RR and gene density and a weak to no correlation between RR and genetic diversity [36, 60, 62]. In the case of *Oryza sativa* [57] and *E. globulus* the strong negative correlation between RR and diversity is likely to reflect, at least in part, the strong correlation between gene density and RR. However, in the case of eucalypts the contribution of other factors, such as their high genetic diversity [19, 27, 29] (see introduction) other facets of genome architecture, and the history of population size within each species, requires further investigation. Further, we cannot completely dismiss the possibly that other mechanisms are involved. For example, diversity *per se* could be affecting RR. It is well known that sequence divergence between individuals can inhibit recombination in adjacent regions of the genome, associated with the DNA mis-match repair system [63, 64] and this process could also contribute to the negative correlation between RR and diversity we observe.

## Conclusions

Comparison of our results with previously published estimates provides evidence for stability in chromosomal level recombination rates across eucalypt species. Together with the previously reported stability in karyotypes, genome synteny/colinearity and the genomic distribution of population diversity and divergence across the globally significant lineages of *Eucalyptus,* our finding bode well for the transfer of genomic information between species within these lineages. We also demonstrate that the differences in RR between chromosomes are negatively correlated with species-wide genetic diversity at the chromosome level, which is opposite to the correlation observed in most other taxa. While our findings may in part reflect the scale of investigation, these insights into the recombination landscape of *Eucalyptus* provide testable hypotheses for future research in forest trees. Future research will investigate whether chromosomal differences in recombination rate are conserved between more distantly related eucalypt species, as well as investigating correlations between RR and genomic attributes at finer genomic scales.

## Methods

### Linkage map construction and comparison with physical map

Recombination rate (RR) was estimated in 10 unrelated genotypes, which were the parents of 5 full-sib pedigrees. These were all inter-provenance crosses and included three F_1_ (each with 183 to 184 individuals) and two ‘outbred F_2_’ (172 and 503 individuals) pedigrees. Together, these pedigrees sampled a diverse section of the natural distribution of *E. globulus* (see Hudson et al. [33], Freeman et al. [65]). All of the pedigrees had been previously used for linkage map construction [33, 65] and the progenies genotyped with diversity array technology ([DArT], DArT P/L Ltd Canberra, Australia) [66, 67] and microsatellite markers as described in Hudson et al. [33] and Freeman et al*.* [65]. To estimate RR in this study, we firstly rebuilt each of the 10 parental linkage maps using a subset of the markers used in the original studies. This was done to maximise the accuracy of RR estimates from the genome-anchored markers. The subset of markers were selected based on having a known and unique physical location on the BRASUZ1 reference genome of *E. grandis* [19] based on basic local alignment search tool (BLAST) searches, and linkage mapping criteria as described below.

For BLAST searches, adaptors were removed from the DArT sequences using Cutadapt V1.2.1 [68], then BlastN searches were performed in the Blastall V2.2.26 suite (alignment length > = 50 bp; e-value = 10^−50^). Two BLAST analyses were conducted. Firstly, an auto-blast amongst all marker sequences was performed to identify redundant markers. Secondly, the physical location of non-redundant markers was determined through a BLAST search against the reference genome. For the latter, BLAST results were classified according to the criteria developed by Salse et al*.* [69]: the cumulative percentage of sequence identity (CIP) and the cumulative alignment percentage (CALP), based on the length of high-scoring segment pairs (HSP) and the sum of all HSP lengths (AL). For each marker, the most plausible position on the BRASUZ1 reference genome was identified based on the CIP value with a maximum CALP (markers with CALP > 200 % were removed) which minimised RR.

Of the non-redundant markers anchored to unique genome positions, four main mapping criteria were used to select the markers used to estimate RR. Ordered by priority, these criteria were: i) genome coverage; ii) segregation; iii) linkage statistics; and iv) RR. Markers with weak linkage statistics were excluded, with the exception of a few markers which were included to maximise genome coverage, provided their map order matched the physical map. Uniparentally inherited markers segregating 1:1 were selected preferentially to biparentally inherited markers segregating 3:1 (preventing auto-correlations when comparing recombination estimates in parental maps from the same pedigree), although markers segregating 3:1 were selected in cases where they increased map coverage. Linkage analysis was performed using the maximum likelihood algorithm in Join-Map v.4 [70]. Map distance in cM was calculated using Haldane’s mapping function. To estimate RR (cM/Mb)*,* we aligned linkage groups (LGs) against the 11 main scaffolds of BRASUZ1. MapChart 2.2 software [71] was used to compare physical and genetic maps. Markers were accepted when the RR between adjacent markers was less than 25 cM/Mb, since greater RR probably reflects genotyping error(s).

### Variation in RR and relationships with genome features and population genetic parameters

RR was estimated by comparing 2,452 meioses (i.e., 1,226 progenies in total across the 5 pedigrees × 2 parents in each pedigree; see above) in *E. globulus* over the 640 Mb of the reference genome of *E. grandis*. Variation in RR was studied at two different levels: within chromosomes and genome-wide. At the chromosome level, two estimates of RR were used: i) a RR for each chromosome of each parent, calculated as the ratio of the total LG length in cM to the corresponding physical size per scaffold in Mb; and ii) an average chromosome RR (*n* = 11) across all 10 parents. The RR for each chromosome of each parent (*n* = 110; Table 2) was used to test the significance of the parent and chromosome main effects, using a fixed effect model with no interaction and Type 3 Sum of Squares. The chromosome averages (*n* =11) were used for studying the (Pearson’s) correlation with other chromosomes features (chromosome average data for the main features are presented in Table 4). These analyses were undertaken using PROC MIXED and PROC CORR of SAS respectively. At the genome-wide level, for each parent, we used the total genetic distance and the corresponding physical size, to estimate an average RR.

Heterozygosity, diversity and divergence indexes were correlated against RR at the chromosome level. Relative heterozygosity was estimated for each individual and at the chromosome level for each individual, from the total number of polymorphic markers derived from the DArT assay. Diversity and divergence indexes were derived from independent samples [34]. These diversity indexes were calculated from six *Eucalyptus* species from subgenus *Symphyomyrtus*, using ~ 90 range-wide samples per species. The six species belong to three sections within *Symphyomyrtus*: section Latoangulatae (*E. grandis* and *E. urophylla*), section Maidenaria (*E. globulus*, *E. nitens* and *E. dunnii*) and section Exsertaria (*E. camaldulensis*). These species are among the most important hardwood plantations species world-wide and are representative of the sections (lineages) that contain the other economically important eucalypt species [29]. We used the average diversity (*H*_*Hw*_) in each species, as well as the average diversity within, and divergence (*F*_*st*_) between, the six species in our correlation analyses at the chromosome level. The ‘global’ estimates (i.e., those averaged across six species) of diversity and divergence were also analysed after partitioning the markers into those within genes and those >5 kb from a gene (i.e., intra- versus intergenic markers; following [34]).

We also tested for relationships between RR in *E. globulus* and genomic features of the *Eucalyptus* reference genome [19], based on our estimates of average RR for each chromosome, using Pearson’s correlation. These chromosomal features included: physical size of each *E. grandis* chromosome scaffold (in Mb), the GC content, average gene density, the number of tandem duplicated genes and the density of transposable elements (also broken into subcategories of DNA transposable elements, retrotransposons and uncategorised elements, and superfamilies within the DNA transposable elements and retrotransposon subcategories).

## Abbreviations

AL, the sum of all HSP lengths; BLAST, basic local alignment search tool; CALP, the cumulative alignment percentage; CIP, the cumulative percentage of sequence identity; cM, centimorgan; CV, coefficient of variation; DArT, diversity array technology; *F*_*st*_, the proportion of the genetic variance in a sub population relative to the total genetic variance; *H*_*Hw*_, genetic diversity assuming Hard-Weinberg equilibrium; HSP, the length of high-scoring segment pairs; LGs, linkage groups; LINE, long interspersed nuclear element; Mb, megabase; QTL, quantitative trait loci; RR, recombination rate; SINE, short interspersed nuclear element
